# Gap analysis on the biology of Mediterranean marine fishes

**DOI:** 10.1371/journal.pone.0175949

**Published:** 2017-04-13

**Authors:** Donna Dimarchopoulou, Konstantinos I. Stergiou, Athanassios C. Tsikliras

**Affiliations:** 1 Laboratory of Ichthyology, Department of Zoology, School of Biology, Aristotle University of Thessaloniki, Thessaloniki, Greece; 2 Institute of Marine Biological Resources and Inland Waters, Hellenic Centre for Marine Research, Athens, Greece; University of Windsor, CANADA

## Abstract

We estimated the current level of knowledge concerning several biological characteristics of the Mediterranean marine fishes by carrying out a gap analysis based on information extracted from the literature, aiming to identify research trends and future needs in the field of Mediterranean fish biology that can be used in stock assessments, ecosystem modeling and fisheries management. Based on the datasets that emerged from the literature review, there is no information on any biological characteristic for 43% (n = 310) of the Mediterranean fish species, whereas for an additional 15% (n = 109) of them there is information about just one characteristic. The gap between current and desired knowledge (defined here as having information on most biological characteristics for at least half of the Mediterranean marine fishes) is smaller in length-weight relationships, which have been studied for 43% of the species, followed by spawning (39%), diet (29%), growth (25%), maturity (24%), lifespan (19%) and fecundity (17%). The gap is larger in natural mortality for which information is very scarce (8%). European hake (*Merluccius merluccius*), red mullet (*Mullus barbatus*), annular seabream (*Diplodus annularis*), common pandora (*Pagellus erythrinus*), European anchovy (*Engraulis encrasicolus*), European pilchard (*Sardina pilchardus*) and bogue (*Boops boops*) were the most studied species, while sharks and rays were among the least studied ones. Only 25 species were fully studied, i.e. there was available information on all their biological characteristics. The knowledge gaps per characteristic varied among the western, central and eastern Mediterranean subregions. The number of available records per species was positively related to total landings, while no relationship emerged with its maximum reported length, trophic level and commercial value. Future research priorities that should be focused on less studied species (e.g. sharks and rays) and mortality/fecundity instead of length-weight relationships, as well as the economy of scientific sampling (using the entire catch to acquire data on as many biological characteristics as possible) are discussed.

## Introduction

Ichthyology and fish biology were born in the Mediterranean Sea with Aristotle (384–322 BC) being the first to describe aspects of the life history of fishes [1] with his observations on the habitat, diet, and spawning of red mullets (*Mullus* spp.) and annular seabream (*Diplodus annularis*), among other species [2]. Aristotle was also the first who identified the trophic level concept [3]. Nevertheless, the Mediterranean Sea is today one of the areas where key biological information is missing for many fish species [4], especially in its southern part [5]. Concerning fisheries, the Mediterranean Sea is also considered as a fisheries data-poor area [6] with the number of stocks routinely assessed being low in terms of landings and number of stocks/species [7]. To that end, a series of reviews have been conducted on the biology of Mediterranean fishes that included a large part of “grey” literature, i.e. information not published in international journal articles but included in technical reports, national and international conference proceedings and dissertations. These review articles covered the feeding habits and trophic levels [8, 9], onset and duration of spawning [10], length [11] and age at maturity [12], growth [13, 14] and fecundity [15] of Mediterranean marine fishes.

A detailed knowledge of biological characteristics (growth, mortality, maturity) of marine fishes and invertebrates is important in age-based models used in stock assessments that provide the basis for decision-making in fisheries management [16]. The biological characteristics are also used in estimating species resilience, which is a function of lifespan, age/size at maturity, growth, and fecundity, used to examine the effect of fishing on exploited marine organisms [17, 18]. Resilience, together with catch data, has recently been used in stock assessment models that are proposed for fisheries data-poor areas [19, 20].

In addition, the ecosystem approach to fisheries management requires that decision making should be based not only on the characteristics of a particular stock, but on all components of the ecosystem [21]. This is because fish species respond differently to reduced fishing pressure and population time to recovery depends upon the life-history strategy and ecological traits [22, 23]. This holistic approach demands, apart from large-scale research on the biological characteristics of commercial species (growth, maturity, spawning, fecundity, mortality, lifespan and diet), studies of regional interest and on a wide number of species including non-commercial ones [24] for many of which information is currently unavailable. It seems though that, in general, regional studies have been considered of low interest for many years and discouraged by major scientific publishers [5], with only few recent exceptions [18], further adding to the gap between current and desired knowledge. Desired knowledge is defined here as having information on most biological characteristics for at least half of the Mediterranean marine fishes.

The objective of the present work was to record the available information on key biological characteristics of the Mediterranean marine fishes, aiming at identifying data gaps in the available knowledge. Also to examine the hypotheses that the number of available records per species depends on the commercial value of the species or its landings and if it is related to its size and position in the marine food web. Finally, to provide some recommendations for future research targets for fisheries science and management based on the actual needs (i.e. information gaps and missing data) and discuss how these targets can be achieved through the maximum economy of scientific sampling. Once gaps are minimized, the ecosystem indicators and species description will be improved and will in turn contribute to the better understanding of their biology and interactions within the ecosystem perspective.

## Materials and methods

**Fig 1:**
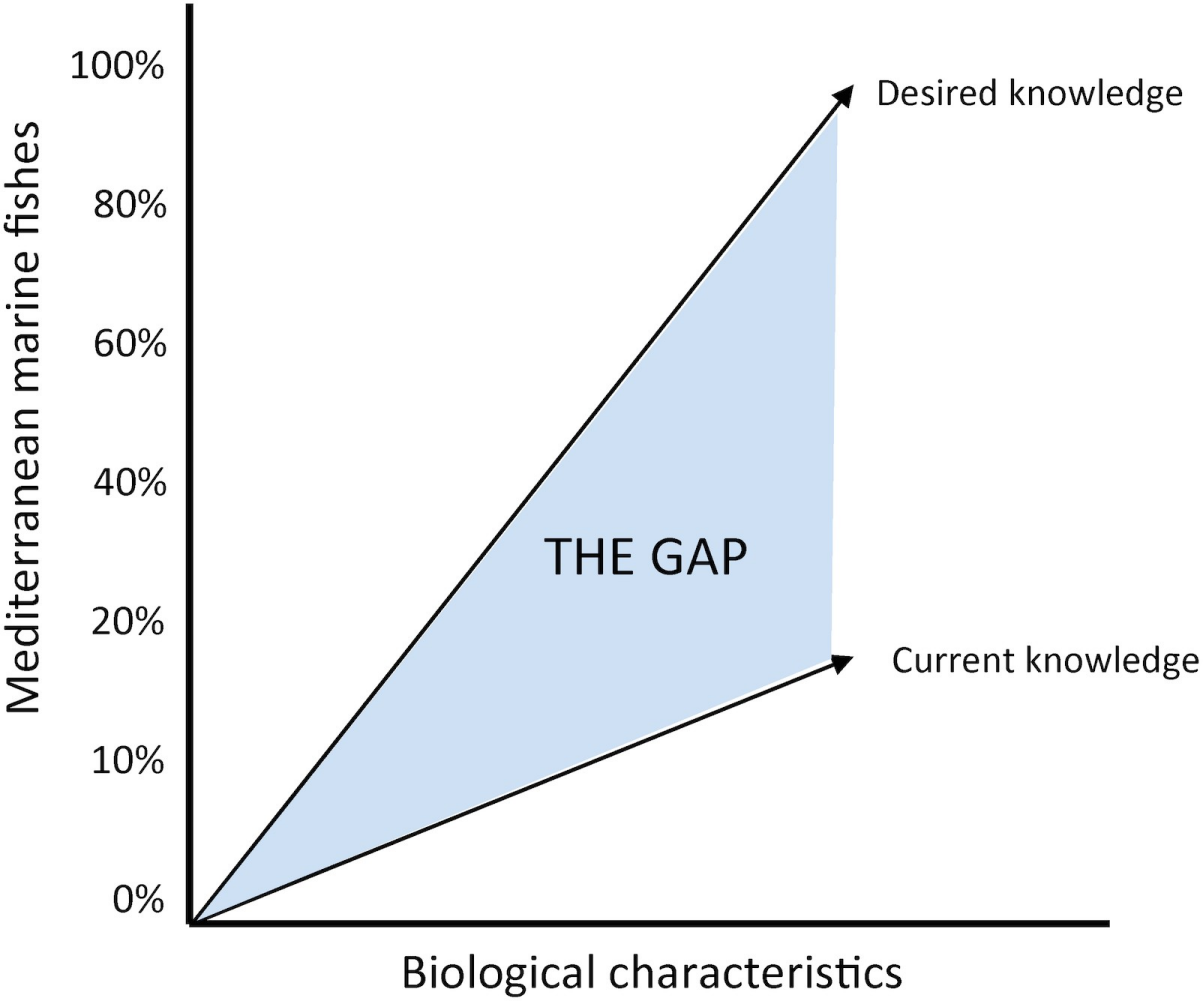
Theoretical plot of gap analysis regarding biological knowledge of the Mediterranean marine fishes.

Gap analysis, as used in management literature (i.e. not ecological gap analysis as defined by [25]), clearly addresses the question "where we are and where we want to be". The analysis makes a comparison of the actual/current performance with the potential/desired one [26] revealing the parts that can be improved towards that desired state (Fig 1). Here, a gap analysis was carried out on Mediterranean marine fishes to investigate the missing biological information, overall, as well as on a subregional basis (W: western; C: central; E: eastern). The gap stems from the difference between current and desired knowledge level; namely the percentage of fish species for which at least one biological characteristic has been studied so far and the ideal situation in which all biological characteristics are known for all species [27], or at least the majority of characteristics for as many species as possible.

We revealed this gap by estimating the proportion of species and biological characteristics that has been fully or partially studied, including the most/least studied species, as well as the most/least studied biological characteristics. We collected data on all fish species that have been recorded in the Mediterranean Sea large marine ecosystem as they are listed in FishBase [28]. Overall, 749 Mediterranean marine fishes are listed in FishBase, of which we excluded the misidentified or questionable records (n = 35 species). For each of the 714 species that were included in the analysis, the available information on age and growth (growth parameters, length-weight relationships, and maximum age), mortality rate, reproduction (spawning, size at maturity, and fecundity) and diet composition was extracted from FishBase and recent reviews on spawning period [10], fecundity [15], size at maturity [11], and feeding [8, 9]. Unpublished or submitted reviews on length-weight relationships and growth parameters were also included along with a thorough literature review of the recent publications that were not included in these reviews. We also estimated the number of new species studied per year (i.e. species for which no information was available before) for the biological characteristics for which such information exists in the aforementioned reviews. For practical purposes, the fish species were categorized as commercial, common non-commercial, protected, and species with atypical life history strategies (e.g. those exhibiting very slow growth or providing parental care to their offspring). The protection status (IUCN Red List of Threatened Species) of each species was also recorded based on the IUCN categories (LC: least concern; EN: endangered; DD: data deficient; NE: not evaluated; NT: near threatened; VU: vulnerable; CR: critically endangered).

For length-weight relationships (LWR) we considered records with both the slope (b) and intercept (a) of the relationship, for somatic growth (G) records with the asymptotic length (L_∞_) and the rate at which it is approached (K), and for lifespan (A) records with the maximum age (t_max_). The onset and duration of spawning (Spawn) and length at maturity (L_m_) were considered to identify spawning and maturity records, respectively, while absolute and relative number of oocytes produced per female were considered for fecundity (Fec). The prey items and feeding preferences were used as records for diet (Diet), whereas for natural mortality (M) the natural mortality rate regardless its estimation method.

To examine whether the number of records per species depends on the species size, position in the food web or commercial value, we also extracted from FishBase the maximum reported length (L_max_) and trophic level (τ) for all species as well as their commercial value (Val), shown as price category in FishBase (VH: very high; H: high; M: medium; L: low). We also extracted their total Mediterranean landings averaged for the last five years (2010–2014) from the FAO-GFCM database [29]. We then cross-correlated the number of records per species with the abovementioned parameters for species with at least one record (n = 404) using Spearman’s correlation coefficient (ρ). Finally, we calculated the number of records per ton of catch (records/t) to allow comparisons between highly commercial and non-commercial species and per cm of somatic length (records/cm) to allow comparisons among sizes.

## Results

**Fig 2:**
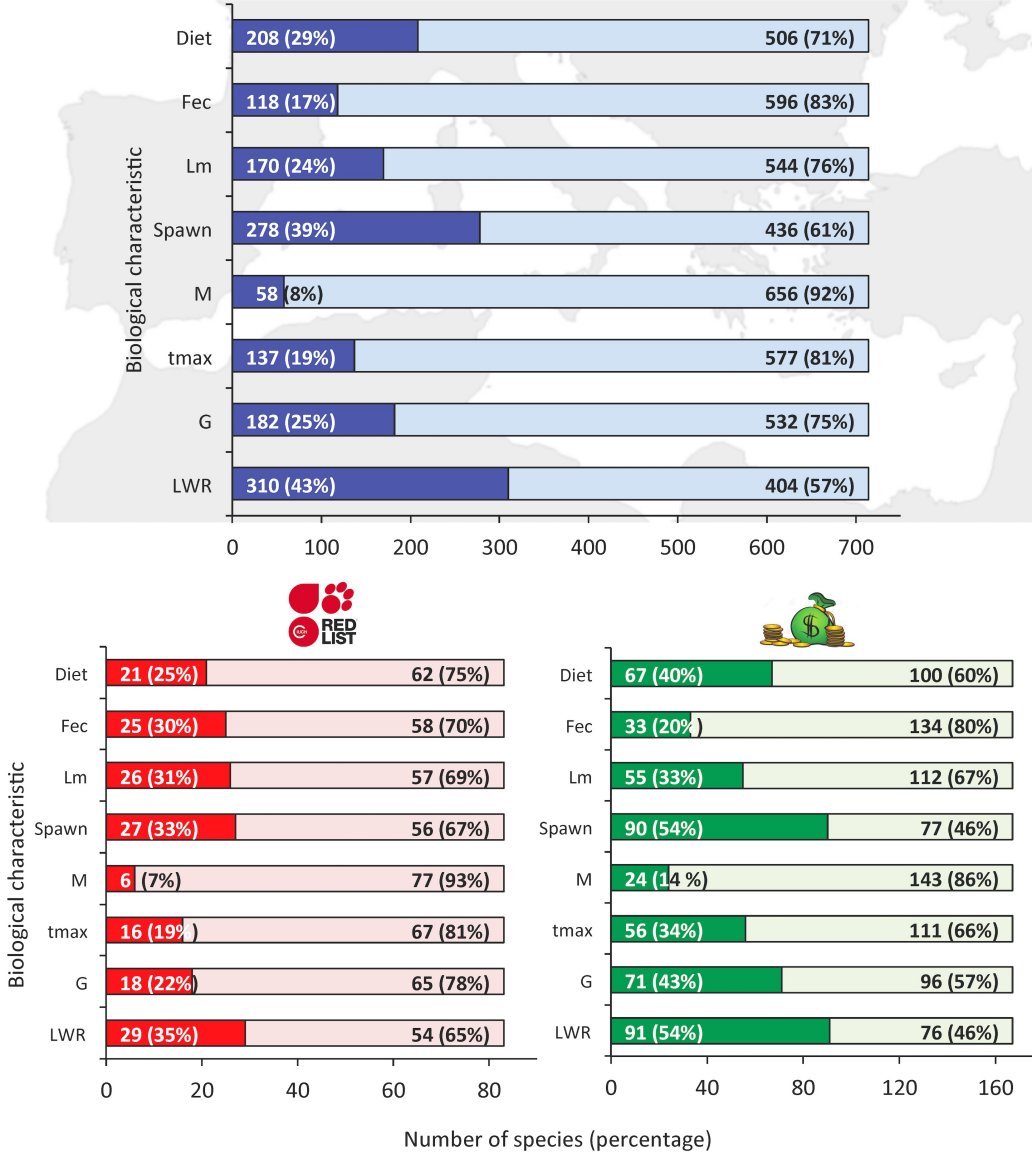
Percentage of Mediterranean fish species with (dark blue) and without (light gray) information on length-weight relationships (LWR), growth parameters (G), maximum age (t_max_), mortality rate (M), spawning period (Spawn), size at maturity (L_m_), feeding preferences (Diet), and fecundity (Fec). The same percentage has also been calculated for species listed in the IUCN Red List of Threatened Species (bottom left panel) under the categories near threatened (NT), vulnerable (VU), endangered (EN) and critically endangered (CR) and those with high (H) and very high (VH) commercial value (bottom right panel).

Based on the assembled dataset of 714 Mediterranean fish species, there is no information for any biological characteristic for 310 species (43%), while for 109 (15%) of them there is information for only one characteristic. As far as the individual biological characteristics are concerned, the gap is smaller for length-weight relationships which are the most common characteristic as they have been studied for 310 (43%) species, followed by spawning (278 species; 39%), diet (208 species; 29%), growth (182 species; 25%), maturity (170 species; 24%), lifespan (137 species; 19%), and fecundity (118 species; 17%). The gap is larger in natural mortality (58 species; 8%) for which information is scarce (Fig 2, top panel).

The percentage of studied species that are listed under the categories near threatened (NT), vulnerable (VU), endangered (EN) and critically endangered (CR) of the IUCN Red List (n = 83) compared to all species listed was lower for all biological characteristics except for maturity and fecundity (Fig 2, bottom left panel). In contrast, the percentage of studied species with high (H) and very high (VH) commercial value (n = 167) compared to all species listed was higher for all characteristics (Fig 2, bottom right panel).

In terms of number of studied characteristics, the most studied Mediterranean fish species (Table 1) were European hake (*Merluccius merluccius*), red mullet (*Mullus barbatus barbatus*), annular seabream (*Diplodus annularis*), common pandora (*Pagellus erythrinus*), European anchovy (*Engraulis encrasicolus*), European pilchard (*Sardina pilchardus*), bogue (*Boops boops*), common two-banded seabream (*Diplodus vulgaris*), tub gurnard (*Chelidonichthys lucerna*), Atlantic horse mackerel (*Trachurus trachurus*), axillary seabream (*Pagellus acarne*), white seabream (*Diplodus sargus sargus*), Mediterranean horse mackerel (*Trachurus mediterraneus*), picarel (*Spicara smaris*), big-scale sand smelt (*Atherina boyeri*), Atlantic bluefin tuna (*Thunnus thynnus*), garfish (*Belone belone*), stargazer (*Uranoscopus scaber*), large-scaled gurnard (*Lepidotrigla cavillone*), brown meagre (*Sciaena umbra*), dusky grouper (*Epinephelus marginatus*), Atlantic chub mackerel (*Scomber colias*), brown ray (*Raja miraletus*), grass goby (*Zosterisessor ophiocephalus*) and transparent goby (*Aphia minuta*). For these twenty-five species there is available information for all the biological characteristics considered in the present work (Table 1); thus, they are considered fully studied in the Mediterranean Sea.

**Table 1:** List of the most studied fish species in the Mediterranean Sea based on the number of studied characteristics (No Char.) and the number of records (No Rec.) per characteristic (G: growth parameters; A: lifespan; LWR: length-weight relationships; L_m_: length at maturity; Spawn: onset and duration of spawning; Fec: fecundity; M: mortality; Diet: feeding preferences). The commercial value (Val) as price category (VH: very high; H: high; M: medium; L: low), the protection status (IUCN) as IUCN Red List status category (LC: least concern; EN: endangered; DD: data deficient; NE: not evaluated; NT: near threatened; VU: vulnerable; CR: critically endangered) and exploitation status (ES) of recent assessments (O: overexploited; S: sustainably exploited; -: not assessed) according to [37, 45] have also been included. A cut-off level was set at 7 characteristics and 30 records, i.e. only species with 7 and 8 studied characteristics and availability of 30 or more records were included.

Family	Species	Common Name	Val	IUCN	ES	No Char.	No Rec.	No. of records per characteristic
Merlucciidae	*Merluccius merluccius*	European hake	H	LC	O	8/8	181	53 G, 53 LWR, 19 Spawn, 18 L_m_, 16 Diet, 9 A, 7 M, 6 Fec
Mullidae	*Mullus barbatus barbatus*	Red mullet	M	LC	O	8/8	129	45 G, 38 LWR, 13 Diet, 9 A, 9 L_m_, 8 Spawn, 5 Fec, 2 M
Sparidae	*Diplodus annularis*	Annular seabream	L	LC	O	8/8	98	48 LWR, 16 Spawn, 12 G, 7 L_m_, 6 A, 4 Diet, 3 Fec, 2 M
Sparidae	*Pagellus erythrinus*	Common pandora	M	LC	O	8/8	98	36 LWR, 16 G, 12 A, 11 Diet, 10 Spawn, 9 L_m_, 3 Fec, 1 M
Engraulidae	*Engraulis encrasicolus*	European anchovy	M	LC	O	8/8	87	31 LWR, 20 G, 11 Spawn, 7 L_m_ 6 A, 6 Diet, 4 Fec, 2 M
Clupeidae	*Sardina pilchardus*	European pilchard	L	LC	O	8/8	82	26 G, 19 LWR, 11 Spawn, 7 A, 7 L_m_, 5 Diet, 4 Fec, 3 M
Sparidae	*Boops boops*	Bogue	H	LC	O	8/8	76	25 LWR, 18 G, 12 Spawn, 7 L_m_, 6 A, 4 Diet, 2 Fec, 2 M
Sparidae	*Diplodus vulgaris*	Common two-banded seabream	L	LC	-	8/8	61	21 LWR, 9 Spawn, 8 G, 7 Diet, 7 L_m_, 5 A, 3 M, 1 Fec
Triglidae	*Chelidonichthys lucerna*	Tub gurnard	M	LC	-	8/8	50	14 LWR, 8 G, 7 Diet, 6 L_m_, 6 Spawn, 5 A, 3 Fec, 1 M
Carangidae	*Trachurus trachurus*	Atlantic horse mackerel	M	VU	S	8/8	48	17 LWR, 7 Diet, 7 G, 7 L_m_, 4 Spawn, 3 A, 2 M, 1 Fec
Sparidae	*Pagellus acarne*	Axillary seabream	M	LC	O	8/8	47	19 LWR, 6 G, 6 L_m_, 5 Spawn, 4 A, 3 Fec, 2 Diet, 2 M
Sparidae	*Diplodus sargus sargus*	White seabream	VH	LC	O	8/8	44	16 LWR, 6 Diet, 6 L_m_, 6 Spawn, 4 G, 3 A, 2 Fec, 1 M
Carangidae	*Trachurus mediterraneus*	Mediterranean horse mackerel	L	LC	O	8/8	43	21 LWR, 7 Diet, 6 G, 4 Spawn, 2 A, 1 Fec, 1 L_m_, 1 M
Sparidae	*Spicara smaris*	Picarel	M	LC	O	8/8	40	19 LWR, 9 G, 3 Diet, 3 Spawn, 2 A, 2 M, 1 Fec, 1 L_m_
Atherinidae	*Atherina boyeri*	Big-scale sand smelt	H	LC	S	8/8	37	15 LWR, 8 Spawn, 5 G, 2 A, 2 Diet, 2 Fec, 2 L_m_, 1 M
Scombridae	*Thunnus thynnus*	Atlantic bluefin tuna	VH	EN	O	8/8	37	10 G, 8 LWR, 6 Spawn, 5 Diet, 3 M, 2 Fec, 2 L_m_, 1 A
Belonidae	*Belone belone*	Garfish	H	LC	O	8/8	31	12 LWR, 5 Spawn, 4 G, 3 Diet, 2 A, 2 Fec, 2 L_m_, 1 M
Uranoscopidae	*Uranoscopus scaber*	Stargazer	U	LC	-	8/8	31	13 LWR, 5 Diet, 3 G, 3 L_m_, 3 Spawn, 2 Fec, 1 A, 1 M
Triglidae	*Lepidotrigla cavillone*	Large-scaled gurnard	L	NE	-	8/8	30	9 LWR, 5 Diet, 5 Spawn, 4 G, 3 L_m_, 2 A, 1 Fec, 1 M
Sciaenidae	*Sciaena umbra*	Brown meagre	VH	NT	-	8/8	28	9 LWR, 7 Spawn, 3 Diet, 3 G, 3 L_m_, 1 A, 1 Fec, 1 M
Serranidae	*Epinephelus marginatus*	Dusky grouper	VH	EN	O	8/8	25	6 Spawn, 5 LWR, 4 L_m_, 3 A, 3 G, 2 Diet, 1 Fec, 1 M
Scombridae	*Scomber colias*	Atlantic chub mackerel	U	LC	O	8/8	25	12 LWR, 4 G, 3 Spawn, 2 A, 1 Diet, 1 Fec, 1 L_m_, 1 M
Rajidae	*Raja miraletus*	Brown ray	M	LC	-	8/8	22	7 LWR, 5 Diet, 3 Spawn, 2 G, 2 L_m_, 1 A, 1 Fec, 1 M
Gobiidae	*Zosterisessor ophiocephalus*	Grass goby	VH	LC	-	8/8	22	7 LWR, 4 Spawn, 3 G, 2 A, 2 Fec, 2 L_m_, 1 Diet, 1 M
Gobiidae	*Aphia minuta*	Transparent goby	VH	NE	-	8/8	13	4 Spawn, 3 LWR, 1 A, 1 Diet, 1 Fec, 1 G, 1 L_m_, 1 M
Mullidae	*Mullus surmuletus*	Surmullet	VH	LC	O	7/8	99	39 LWR, 16 G, 16 Spawn, 12 Diet, 7 L_m_, 6 A, 3 M
Serranidae	*Serranus cabrilla*	Comber	M	LC	-	7/8	59	28 LWR, 9 Spawn, 6 Diet, 6 G, 5 A, 4 L_m_, 1 M
Scorpaenidae	*Scorpaena porcus*	Black scorpionfish	L	LC	-	7/8	57	24 LWR, 11 Diet, 7 Spawn, 5 G, 5 L_m_, 4 A, 1 Fec
Clupeidae	*Sardinella aurita*	Round sardinella	M	LC	O	7/8	53	17 G, 12 LWR, 6 L_m_, 6 Spawn, 5 Diet, 4 Fec, 3 A
Sparidae	*Sparus aurata*	Gilthead seabream	VH	LC	-	7/8	49	21 LWR, 10 G, 6 Spawn, 4 A, 4 L_m_, 3 M, 1 Diet
Sparidae	*Lithognathus mormyrus*	Sand steenbras	M	LC	-	7/8	46	22 LWR, 6 L_m_, 6 Spawn, 5 G, 4 Diet, 2 A, 1 M
Sparidae	*Spicara maena*	Blotched picarel	H	LC	O	7/8	42	18 LWR, 8 G, 5 Diet, 4 L_m_, 4 Spawn, 2 A, 1 Fec
Mugilidae	*Liza aurata*	Golden grey mullet	M	LC	-	7/8	41	18 LWR, 10 G, 4 A, 3 Spawn, 2 Fec, 2 L_m_, 2 M
Moronidae	*Dicentrarchus labrax*	European seabass	VH	LC	O	7/8	39	16 LWR, 8 Spawn, 6 G, 3 Fec, 3 L_m_, 2 A, 1 M
Mugilidae	*Liza ramada*	Thinlip grey mullet	M	LC	-	7/8	35	11 LWR, 7 L_m_, 5 Fec, 5 G, 5 Spawn, 1 A, 1 M
Gadidae	*Micromesistius poutassou*	Blue whiting	L	NE	O	7/8	32	10 G, 10 LWR, 4 Diet, 3 Spawn, 2 A, 2 L_m_, 1 M
Scorpaenidae	*Scorpaena notata*	Small red scorpionfish	L	LC	-	7/8	32	15 LWR, 6 Diet, 3 G, 3 Spawn, 2 A, 2 L_m_, 1 Fec

In terms of number of records, the most studied Mediterranean fish species (Table 1) were European hake (181 records in total), red mullet (129 records in total), surmullet (*Mullus surmuletus*) (information for all characteristics but fecundity; 99 records in total), annular seabream (98 records in total), common pandora (98 records in total), European anchovy (87 records in total), European pilchard (82 records in total) and bogue (76 records in total).

**Fig 3:**
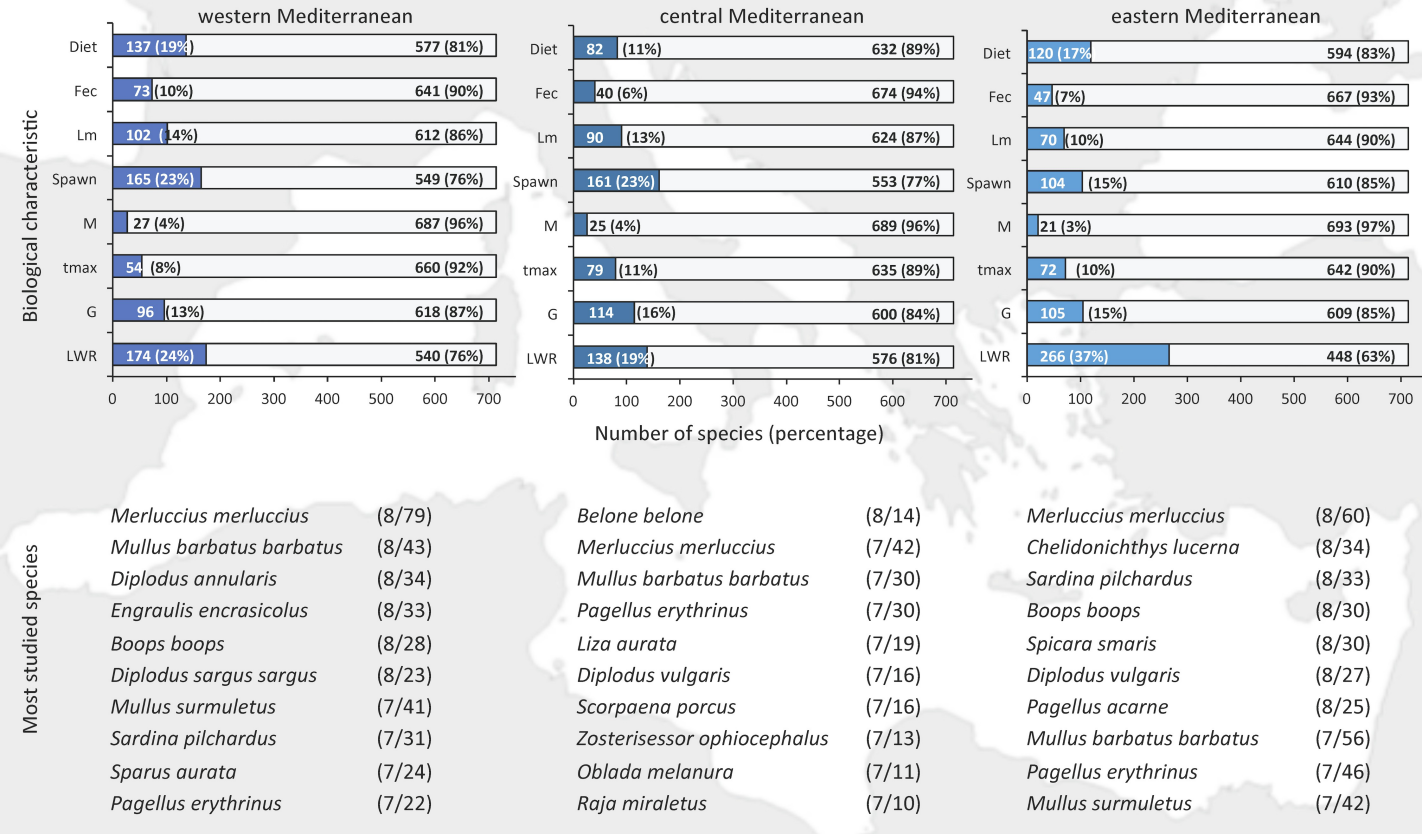
Percentage of fish species with (blue) and without (gray) information on length-weight relationships (LWR), growth parameters (G), maximum age (t_max_), mortality rate (M), spawning period (Spawn), size at maturity (L_m_), feeding preferences (Diet), and fecundity (Fec) across the western, central and eastern Mediterranean. The ten most studied species are also listed per area (number of characteristics/number of records).

Although the general pattern on the studied characteristics and the most studied species holds among the western, central and eastern Mediterranean, there are some spatial variations within each biological characteristic (Fig 3). In the eastern and western Mediterranean the percentage of studied species is higher (41 and 40%, respectively), compared to the central subregion where there is information for at least one biological characteristic for 34% of the species. Diet was most extensively studied in the western and eastern subregion, fecundity in the western, maturity and spawning in the western and central, lifespan and growth in the central and eastern, and LWR was mostly studied in the eastern subregion (Fig 3). Mortality records were rather balanced among subregions (Fig 3). Hake, red mullet, and common pandora were among the most studied species across subareas, with hake being the top studied species in all subareas in terms of records (Fig 3).

Our analysis showed that a large number of highly commercial fishes were understudied with available information on less than half of their biological characteristics (Table 2). Several commercial serranids (Family Serranidae), triglids (Family Triglidae), labrids (Family Labridae) and flatfishes (families Soleidae, Bothidae and Pleuronectidae) were included in the list of the least studied commercial fishes (Table 2). Among the least studied species were also most sharks and rays, especially the rare and large ones such as basking shark (*Cetorhinus maximus*), thresher (*Alopias vulpinus*), and great white shark (*Carcharodon carcharias*). Among the least studied rays were also the stingrays of the family Dasyatidae, and most skates and rays of the family Rajidae, while among the least studies sharks were the members of the families Hexanchidae and Squatinidae. Finfishes with atypical reproductive strategies, such as most gobies (Family Gobiidae) and pipefish (Family Syngnathidae) (Table 2). There is missing information for the mesopelagic lantern fishes (Family Myctophidae) most of which have not been studied at all. Even the opah (*Lampris guttatus*), the first known warm-blooded fish [30], and the ocean sunfish (*Mola mola*), which is the most fecund fish with each female producing over 300 million eggs per spawning act [31], have not been studied at all in the Mediterranean Sea. Key biological information was also missing for species protected by law and international conservation conventions (Table 2), such as the blue shark (*Prionace glauca*) and devil fish (*Mobula mobular*).

**Table 2:** List of some of the least studied fish species in the Mediterranean Sea based on the number of studied characteristics and the number of records per characteristic (G: growth parameters; A: lifespan; LWR: length-weight relationships; L_m_: length at maturity; Spawn: onset and duration of spawning; Fec: fecundity; M: mortality; Diet: feeding preferences). The commercial value (Val) as price category (VH: very high; H: high; M: medium; L: low; U: unknown), the protection status (IUCN) as IUCN Red List status category (LC: least concern; EN: endangered; DD: data deficient; NE: not evaluated; NT: near threatened; VU: vulnerable; CR: critically endangered) and exploitation status (ES) of recent assessments (O: overexploited; S: sustainably exploited; -: not assessed) according to [37, 45] have also been included.

Family	Species	Common Name	Val	IUCN	ES	No. of studied characteristics	No. of records	No. of records/characteristic
**Species with commercial value**								
Bothidae	*Arnoglossus thori*	Thor’s scaldfish	VH	DD	-	3/8	9	6 LWR, 2 Diet, 1 Spawn
Pleuronectidae	*Platichthys flesus*	European flounder	VH	LC	-	3/8	8	4 LWR, 2 G, 2 L_m_
Triglidae	*Chelidonichthys obscurus*	Longfin gurnard	VH	NE	-	3/8	7	4 Diet, 2 LWR, 1 Spawn
Serranidae	*Epinephelus costae*	Goldblotch grouper	VH	DD	-	3/8	7	4 LWR, 2 G, 1 Diet
Gobiidae	*Gobius cobitis*	Giant goby	VH	NE	-	3/8	7	3 A, 2 LWR, 2 Spawn
Soleidae	*Microchirus variegatus*	Thickback sole	VH	LC	-	3/8	6	3 Spawn, 2 LWR, 1 Diet
Cynoglossidae	*Symphurus nigrescens*	Tonguesole	VH	LC	-	3/8	6	3 LWR, 2 Diet, 1 Spawn
Sciaenidae	*Argyrosomus regius*	Meagre	M	LC	-	3/8	5	3 Spawn, 1 G, 1 L_m_
Serranidae	*Anthias anthias*	Swallowtail seaperch	VH	LC	-	3/8	3	1 Diet, 1 LWR, 1 Spawn
Soleidae	*Bathysolea profundicola*	Deepwater sole	VH	LC	-	3/8	3	1 G, 1 L_m_, 1 Spawn
Triakidae	*Galeorhinus galeus*	Tope shark	M	VU	-	3/8	3	1 Fec, 1 G, 1 L_m_
Soleidae	*Microchirus ocellatus*	Foureyed sole	VH	DD	-	2/8	3	2 LWR, 1 Spawn
Triglidae	*Lepidotrigla dieuzeidei*	Spiny gurnard	VH	LC	-	1/8	1	1 LWR
Labridae	*Thallasoma pavo*	Ornate wrasse	VH	LC	-	1/8	1	1 Spawn
Centrolophidae	*Centrolophus niger*	Rudderfish	VH	LC	-	0	0	
Carangidae	*Pseudocaranx dentex*	White trevally	VH	LC	-	0	0	
**Common non-commercial**								
Blenniidae	*Blennius ocellaris*	Butterfly blenny	U	LC	-	2/8	8	6 LWR, 2 Spawn
Callionymidae	*Callionymus lyra*	Dragonet	U	LC	-	2/8	2	1 LWR, 1 Spawn
**Atypical life strategies**								
Syngnathidae	*Syngnathus typhle*	Broadnosed pipefish	U	LC	-	3/8	14	11 LWR, 2 Diet, 1 Spawn
Syngnathidae	*Syngnathus abaster*	Black-striped pipefish	U	LC	-	3/8	10	7 LWR, 2 Spawn, 1 Diet
Syngnathidae	*Hippocampus hippocampus*	Short snouted seahorse	U	DD	-	3/8	7	4 LWR, 2 Diet, 1 Spawn
Dasyatidae	*Dasyatis centroura*	Roughtail stingray	L	LC	-	3/8	3	1 Fec, 1 LWR, 1 Spawn
Dasyatidae	*Dasyatis marmorata*	Marbled stingray	U	DD	-	3/8	3	1 Diet, 1 Fec, 1 L_m_
Dasyatidae	*Dasyatis tortonesei*	Tortonese's stingray	U	NE	-	3/8	3	1 Fec, 1 L_m_, 1 LWR
Hexanchidae	*Heptranchias perlo*	Sharpnose sevengill shark	U	NT	-	3/8	3	1 Fec, 1 L_m_, 1 LWR
Squatinidae	*Squatina aculeata*	Sawback angelshark	M	CR	-	3/8	3	1 Fec, 1 L_m_, 1 Spawn
Squatinidae	*Squatina squatina*	Angelshark	M	CR	-	3/8	3	1 Fec, 1 L_m_, 1 Spawn
Gasterosteidae	*Gasterosteus aculeatus*	Three-spined stickleback	U	LC	-	2/8	2	1 LWR, 1 Spawn
Rajidae	*Leucoraja melitensis*	Maltese ray	U	CR	-	2/8	2	1 Fec, 1 Spawn
Squatinidae	*Squatina oculata*	Smoothback angelshark	M	CR	-	2/8	2	1 Fec, 1 Spawn
Myliobatidae	*Pteromylaeus bovinus*	Bull ray	M	DD	-	1/8	1	1 Fec
Alopiidae	*Alopias superciliosus*	Bigeye thresher	L	VU	-	0	0	
Alopiidae	*Alopias vulpinus*	Thresher	H	VU	-	0	0	
Lampridae	*Lampris guttatus*	Opah	VH	LC	-	0	0	
Molidae	*Mola mola*	Ocean sunfish	U	VU	-	0	0	
Petromyzontidae	*Petromyzon marinus*	Sea lamprey	H	LC	-	0	0	
Scorpaenidae	*Pterois miles*	Devil firefish	U	NE	-	0	0	
Gobiidae	All species		-	-	-			
Myctophidae	All species		-	-	-			
Rajidae	All species		-	-	-			
**Protected**								
Carcharhinidae	*Prionace glauca*	Blue shark	M	NT	-	3/8	5	2 A, 2 G, 1 Spawn
Lamnidae	*Carcharodon carcharias*	Great white shark	L	VU	-	0	0	
Cetorhinidae	*Cetorhinus maximus*	Basking shark	L	VU	-	0	0	
Lamnidae	*Isurus oxyrinchus*	Shortfin mako	M	VU	-	0	0	
Lamnidae	*Lamna nasus*	Porbeagle	M	VU	-	0	0	
Myliobatidae	*Mobula mobular*	Devil fish	U	EN	-	0	0	
**All sharks and rays**								

**Fig 4:**
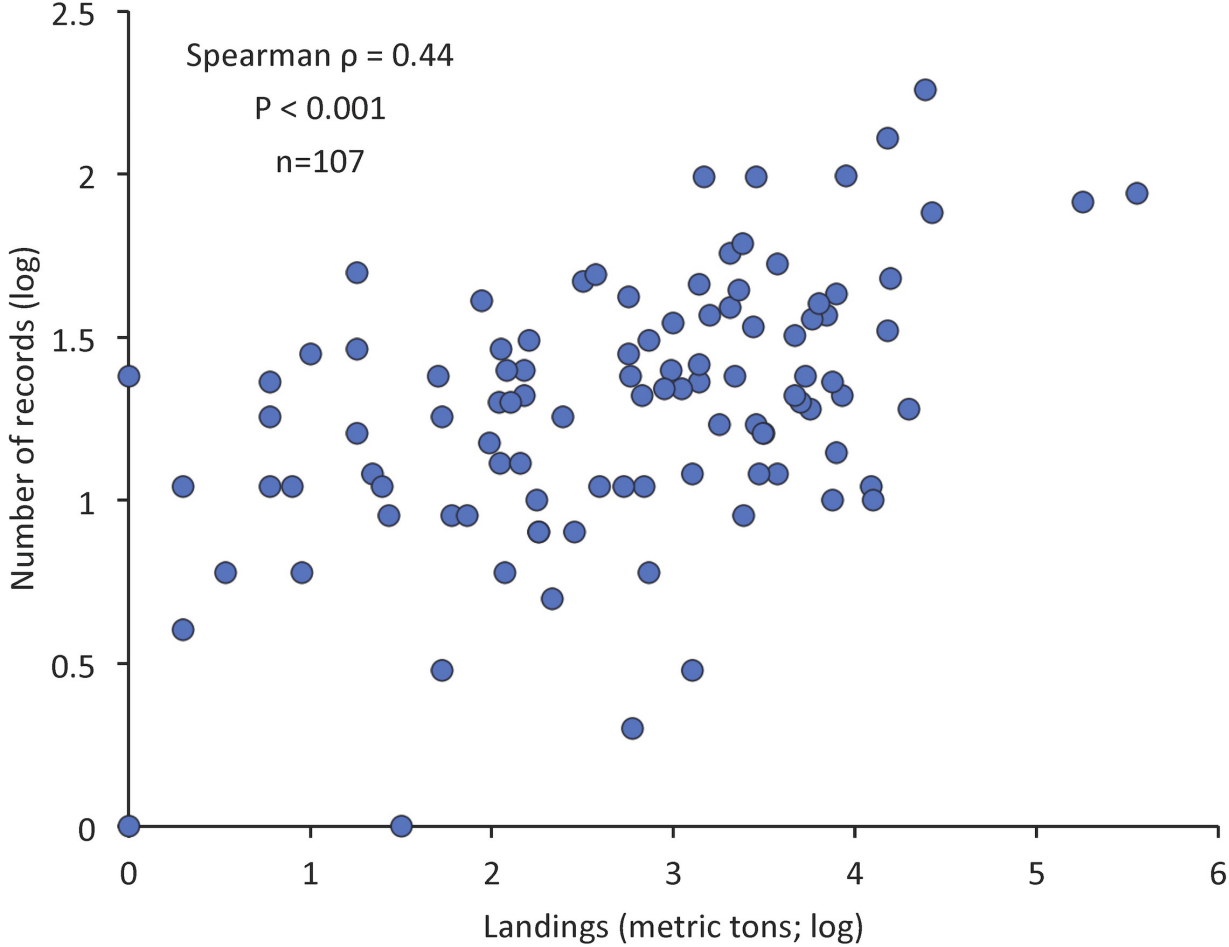
The relationship between number of records and total landings (in metric tons) for Mediterranean fish species with at least one studied biological characteristic and with available landings (n = 107). Both variables were logarithmically transformed to improve visibility of the figure.

For Mediterranean fish species with at least one studied biological characteristic and with available landings data, the number of records was positively correlated to total landings (n = 107, Spearman ρ = 0.44, P < 0.001) (Fig 4). In contrast, no relationship was observed for the number of records with the maximum reported length (n = 404, Spearman ρ = 0.046, P = 0.21), trophic level (n = 404, Spearman ρ < 0.010, P = 0.48) and commercial value (n = 216, Spearman ρ = -0.010, P = 0.88).

The highest number of records per ton of production (records/t) was observed for the black goby (*Gobius niger*) and bluespotted cornetfish (*Fistularia commersonii*), and the highest number of records per cm of somatic length (records/cm) was observed for the annular seabream (4.08 records/cm), anchovy (3.56 records/cm), red mullet (3.18 records/cm), Mediterranean banded killifish (*Aphanius fasciatus*) (3.00 records/cm), European pilchard (2.44 records/cm), and giant goby (*Gobius cobitis*) (2.33 records/cm).

**Fig 5:**
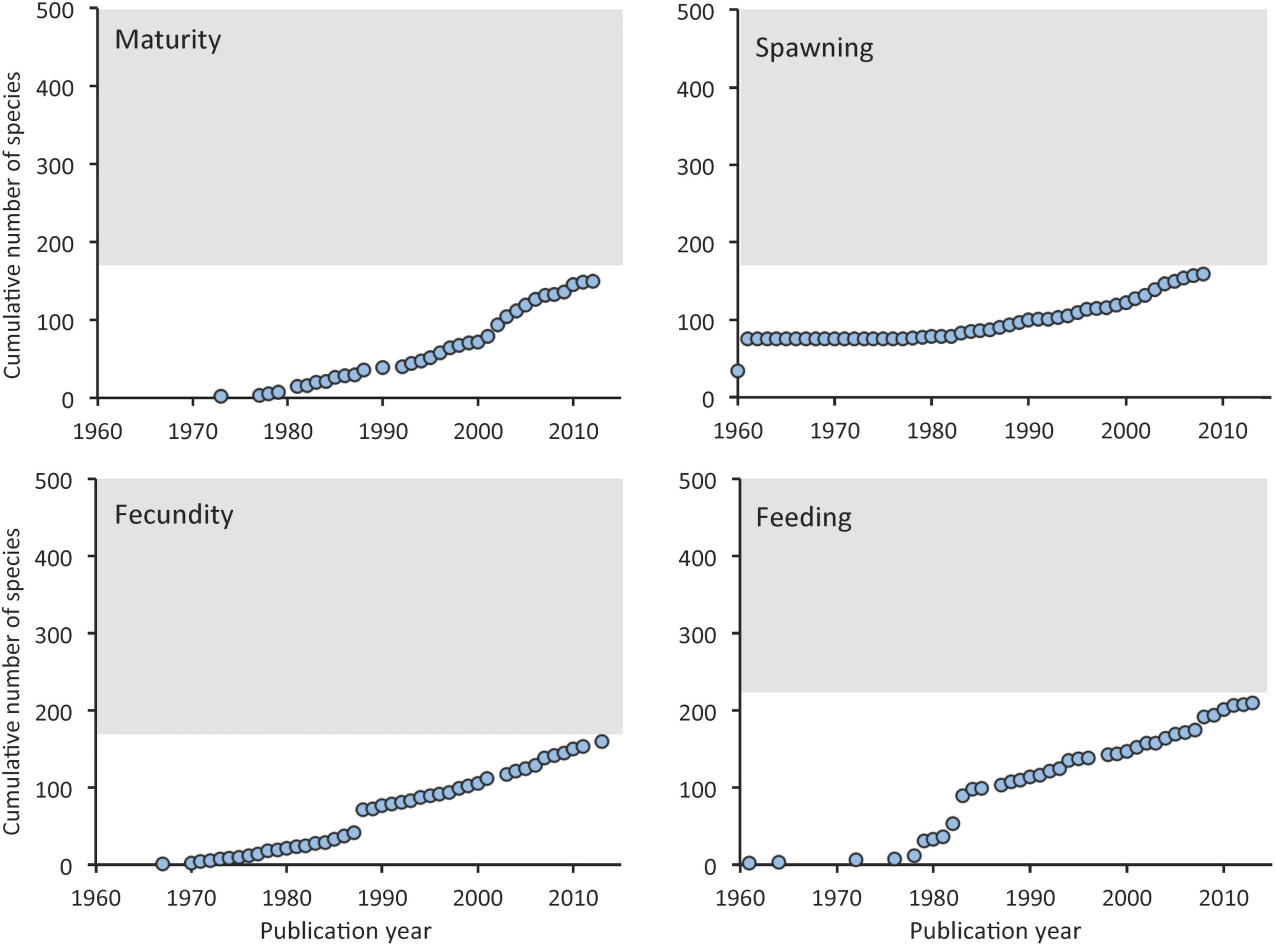
Cumulative number of marine fish species studied with respect to maturity, fecundity, spawning and feeding in the Mediterranean Sea since 1960. The shaded area indicates the knowledge gap to the feasible target of 500 species (see text for details).

The number of new species studied with respect to their spawning, maturity, fecundity and feeding increases with a rate of 2 to 5 new species per year (Fig 5), with the rate of increase being higher for feeding (4.6 per year) followed by maturity (4.2 per year) and fecundity (3.8 per year) and then by spawning (2 per year). After 50 years of research on the biological characteristics of the Mediterranean marine fishes, about 150–212 species (*ca*. 21–30%) have been studied per characteristic but an asymptote has not been reached yet (Fig 5).

## Discussion

Our analysis shows that the majority of the studies focus on fish species of high commercial interest and specifically on length-weight relationships, while at the same time a high proportion of the Mediterranean fishes and important biological characteristics are being neglected [24]. It was quite expected that the biological characteristics that require the least research effort and time and have low cost, such as the length-weight relationships [32, 33], would be the most studied ones and that those that expand over a year cycle with intense sampling (e.g. onset and duration of spawning), require computational analysis (e.g. maturity), specialized equipment (e.g. growth and fecundity), specialized skills (e.g. diet) as well as trained staff with expertise, would be less studied.

It is odd that the maximum age is missing from a relatively large proportion of studies focusing on ageing and growth (Fig 2). The maximum age and natural mortality of a population should be reported in all articles on growth because maximum age is measured anyway and natural mortality can be easily and empirically estimated from growth parameters and sea temperature [34, 35]. Similarly, although determining fish fecundity is a time and cost consuming method requiring staff with expertise, the sampling effort required for collecting the gonads is low in terms of specimens and time [15]. Thus, the study of fecundity could be expanded to cover a larger number of species, especially when one has to record the stages of maturity during the spawning period of the species anyway [36].

There is generally less information on protected species compared to those with high commercial value (Fig 2). Species with high commercial value are more likely to be studied because of their economic importance to the fisheries and because they are usually the “target species” in scientific surveys. They are also the ones that suffer the highest exploitation (Table 1) and those that are being assessed on a more regular basis at stock level [37]. In contrast, the majority of protected species are sharks and rays that are generally understudied partly because the accessibility to samples is confined.

The most studied Mediterranean fish species (Table 1), as shown by our gap analysis, were all commercial and heavily exploited across the Mediterranean [37]. European hake (*Merluccius merluccius*), European pilchard (*Sardina pilchardus*), annular seabream (*Diplodus annularis*) and common pandora (*Pagellus erythrinus*), but also surmullet (*Mullus surmuletus*) and red mullet (*Mullus barbatus barbatus*), belong to the most well-studied fish families in the Mediterranean [11]. Because of their commercial importance and exploitation from the majority of the fleet and gears, most of these species, along with pink shrimp (*Parapeneaus longirostris*) and Norway lobster (*Nephrops norvegicus*) are among the stocks that are routinely assessed in many Mediterranean areas [37]. Red mullet, European hake, surmullet, and common pandora have been also reported as the most studied fish species in a similar research in Greek waters [38].

The knowledge gaps vary among regions but the general pattern of studied characteristics and species per subregion is similar to the total, with length-weight relationships and spawning being the best studied features and hake, red mullet, and common pandora, being the best studied species (Fig 3). The remarkable reporting of length-weight relationships from Turkey during the last decade has increased the contribution of this characteristic in the eastern Mediterranean compared to the other subregions. In a similar work for the marine fish species in the Greek seas, it was shown that 74% of the species had not been studied at all, while for 12% only one characteristic had been studied; length-weight relationships and growth were the most studied characteristics in the Greek seas [38].

Worldwide, Salmonidae is the most frequently and well studied family (contrary to our results because the marine species of this family are not inhabiting the Mediterranean Sea), whereas Atlantic cod (*Gadus morhua*) and European pilchard are the most studied species [39]. Recently, it has been reported that the concentration of research effort to a very limited number of species is a global trend and the publication output is not related to the commercial importance of the species [40], neither in terms of catch, nor in terms of value. The findings of the present work confirm the conclusions of [40] with respect to the lack of any correlation between scientific output and commercial value and the limited published information for a large number of commercially important species. However, when all species not studied at all were excluded from the analysis (i.e. those with zero records), the number of records was positively correlated with the landings (Fig 4), indicating that catch quantity positively affects publication output on the Mediterranean marine fishes.

The presence of black goby (*Gobius niger*) and of the lessepsian bluespotted cornetfish (*Fistularia commersonii*) as the most studied species per ton of catch can be explained if part of the catch of these species remains unreported or aggregated [29], as it is the case for many exotic species. The presence of the Mediterranean banded killifish (*Aphanius fasciatus*) and giant goby (*Gobius cobitis*) among the most studied species per cm of somatic length is certainly surprising and unexpected, but confirms the occasional scientific interest towards species with no commercial value but with atypical behavioural or biological characteristics. In the case of the Mediterranean banded killifish, which is protected by international conventions, one PhD thesis [41] and the articles derived from it was enough for a nearly complete description of its biological characteristics.

Our results are in general agreement with those of [40] and point out that the level of scientific effort devoted to some economically important and most protected species is low. This demonstrates the usefulness of a gap analysis for informing the scientific community, funding agencies, and policy-makers when designing or funding scientific projects. Primary research on fish biology should be encouraged [18], as the higher the knowledge on species inhabiting the Mediterranean Sea, the better the understanding of this complex ecosystem [42] and therefore the more realistic and effective the fisheries management plans that can be developed and implemented.

Although the cumulative number of new marine fish species studied per year in the Mediterranean increases, it has not yet reached an asymptote, which implies that there are still many species to be studied, at least regarding their reproductive biology and feeding (Fig 5). The cumulative number of items published per year on growth, spawning and feeding of Mediterranean fishes has constantly been increasing since the 1960s, with different increasing trends among them [4]. The same increasing trend holds for the number of studied species (Fig 5), indicating that some of the new publications refer to species that had never been studied before [4]. The ideal (though utopian) situation would be when there will be information on the biological characteristics of all Mediterranean species. Yet, some of the species are rare or inaccessible by most fishing gears [43] or even caught in very low quantities that cannot support a study on any biological characteristic [36]. Thus, a reasonable aim of having information on most biological characteristics for 50% of the Mediterranean marine fishes could be reached within 10 to 20 years.

We believe that future research priorities should focus on the biological characteristics for which the gap from desired knowledge is large, especially on mortality and fecundity, whereas length-weight relationships should be studied only for species with no data in FishBase [28]. Concerning the species, we suggest that future research, including postgraduate and doctoral dissertations, should focus on non-commercial, protected, and underexploited species with atypical or slow life history strategies. These priorities should be set within the scope of collecting all the data required for stock assessments and ecosystem management while taking into account important changes in fish life history traits as a response to fishing [22].

Based on the above, sharks and rays that are long-lived, slow growing, late maturing, produce only a few oocytes and are therefore more susceptible to overfishing [44], are among the top priority species to be studied. This does not imply that protected species or those with slow life history strategies should be targeted by researchers just to get their length and weight records. Ideally, the survey or commercial catch should be fully used aiming at the lowest possible waste of biological material, thus ensuring the maximum economy of sampling. When protected species or slow life history strategy species are caught, their specimens should be exhaustively studied across their biological characteristics. It is a complete waste of resources if valuable samples remain unused.
